# The angle of arrival estimation of frequency-hopping cooperative object based on software-defined radio

**DOI:** 10.1038/s41598-024-58488-8

**Published:** 2024-04-02

**Authors:** Rustamaji, Sri Kliwati, Wahyu Widada

**Affiliations:** Research Center for Structural Strength Technology, National Research and Innovation Agency, South Tangerang, 15314 Indonesia

**Keywords:** Cooperative object, Hopping frequency, Bandpass Fourier filter, Azimuth AOA, SDR, Engineering, Electrical and electronic engineering

## Abstract

The primary objective of this research is to propose and examine a technique for accurately determining the azimuth angle of cooperative objects. The proposed methodology aims were to achieve fast processing, increased precision, and enhanced safety. A transmitter emitting a continuous wave with a hopping frequency was utilized in combination with interferometry techniques to measure the angle of arrival (AOA) between two antennas. The efficient method incorporates a coarse-to-fine strategy that improves processing speed and azimuth angle accuracy and to effectively eliminate any signal interference, a Fourier bandpass filter is utilized. The coarse estimation is performed using the fast Fourier transform (FFT) algorithm, while the fine estimation is achieved using the multiple signal classification (MUSIC) algorithm. The fine estimation involves utilizing coarse angle input and a scan limit of 2.5° that is determined from the largest simulated root mean square error (RMSE) value. Modelling and outdoor testing using software defined radio (SDR) have been carried out to assess the proposed methodology. The results from the analysis indicate that the proposed method produces the desired RMSE estimation of less than 1°, thereby validating its accuracy and effectiveness.

## Introduction

Geolocating targets or objects has emerged as a significant concern in recent times, encompassing a variety of domains, including civilian (such as government, scientific, and commercial) and military applications^1^. The tracked object position can be dynamic or static, singular or multiple, enabling the tracking of both friendly and hostile objects. Determining an object's position involves considering essential parameters like distance, angle, and velocity. An illustrative instance of civilian application is the usage of motor vehicle radar to ascertain the direction of nearby vehicles^2^. Numerous researchers have put forth similar research applications, such as employing multi-vehicles to locate enemy radar, enabling smart vehicles, and adopting hybrid methods like AOA and Time of Arrival (TOA) to detect targets^3–5^. Precisely estimating frequency-hopping signal parameters holds utmost importance in Frequency Hopping Spread Spectrum (FHSS) communications, which find extensive use in both military and civilian communications owing to their excellent confidentiality, robust anti-jamming capabilities, efficient networking abilities, and resilience against frequency-selective fading^6^.

Various authors have developed various techniques to measure the AOA. These techniques encompass machine learning-based approaches, the self-initiating MUSIC method, a switched beam system employing simple cross-correlation, and time reversal for detecting low-angle targets^7–11^. These methods usually employ complex antenna arrays and involve extensive processing time. Another approach to measure the AOA angle is by calculating the phase difference between two antennas. Several methods, such as FFT and Hilbert transform, can be utilized to determine the phase difference^12–17^. It is crucial to ensure safety in tracking, which necessitates the separation of the target signal from any interfering signals. To achieve this, researchers commonly employ techniques such as Independent Component Analysis (ICA) and frequency filtering^18,19^. SDR, known for its flexible configuration, affordability, and speed, is frequently utilized in various radio applications. These applications include communication network research, data telemetry applications, and AOA measurement applications^20–23^.

The main objective of this proposed research was to monitor and keep track of the azimuth angle of cooperative entities (referred to as “friends”) in a manner that ensures their safety from any interference or jamming. The proposed method is achieved by utilizing FHSS communications. The transmitter was emitting a known hopping frequency. To effectively and efficiently isolate the desired frequency from any potential interfering frequencies, it is more suitable to employ frequency filtering. This research focuses on the development and examination of an azimuth angle detector that employs software-defined radio. In particular, the utilization of Ettus B210 Universal Software Radio Peripheral (USRP) and Nuand Blade RF 2.0^24,25^ is thoroughly discussed. The main contribution of this research paper is to present a solution to the problem of insecure tracking of friendly objects, which is caused by interference. The proposed solution involves a secure tracking method for friendly objects that utilizes frequency-hopping and employs filtering techniques to separate the desired frequency from interfering frequencies. The process involves a predetermined hopping pattern and carrier frequency selection, which determines the sequence and timing of frequency changes. The strongest signal was chosen if it aligns with the target object's frequencies, and the remaining frequencies are eliminated using a Fourier bandpass filter. In order to achieve accurate AOA estimation results, the FFT algorithm was selected for its quick time consumption. Additionally, the MUSIC algorithm was subsequently employed to search for angles and obtain more precise results locally. This choice of algorithms allowed for a simple method that utilizes only two antennas, ensuring real-time results with a fast processing time and minimal cost. The research conducted simulations with various Signal-to-Noise Ratio (SNR) parameters to validate the effectiveness of the proposed method, comparing them against accuracy measures. Additionally, practical experiments were carried out using SDR. These experiments were performed outdoors, with a clear line of sight, to minimize any interference caused by reflected signals. The proposed method is explained in detail in “Method” section of the paper, while “Simulation and experiment results” section focuses on the simulation and experimentation process, examining the effects of different parameters. Finally, “Conclusion” section provides a concise summary of the research findings.

## Method

**Figure 1:**
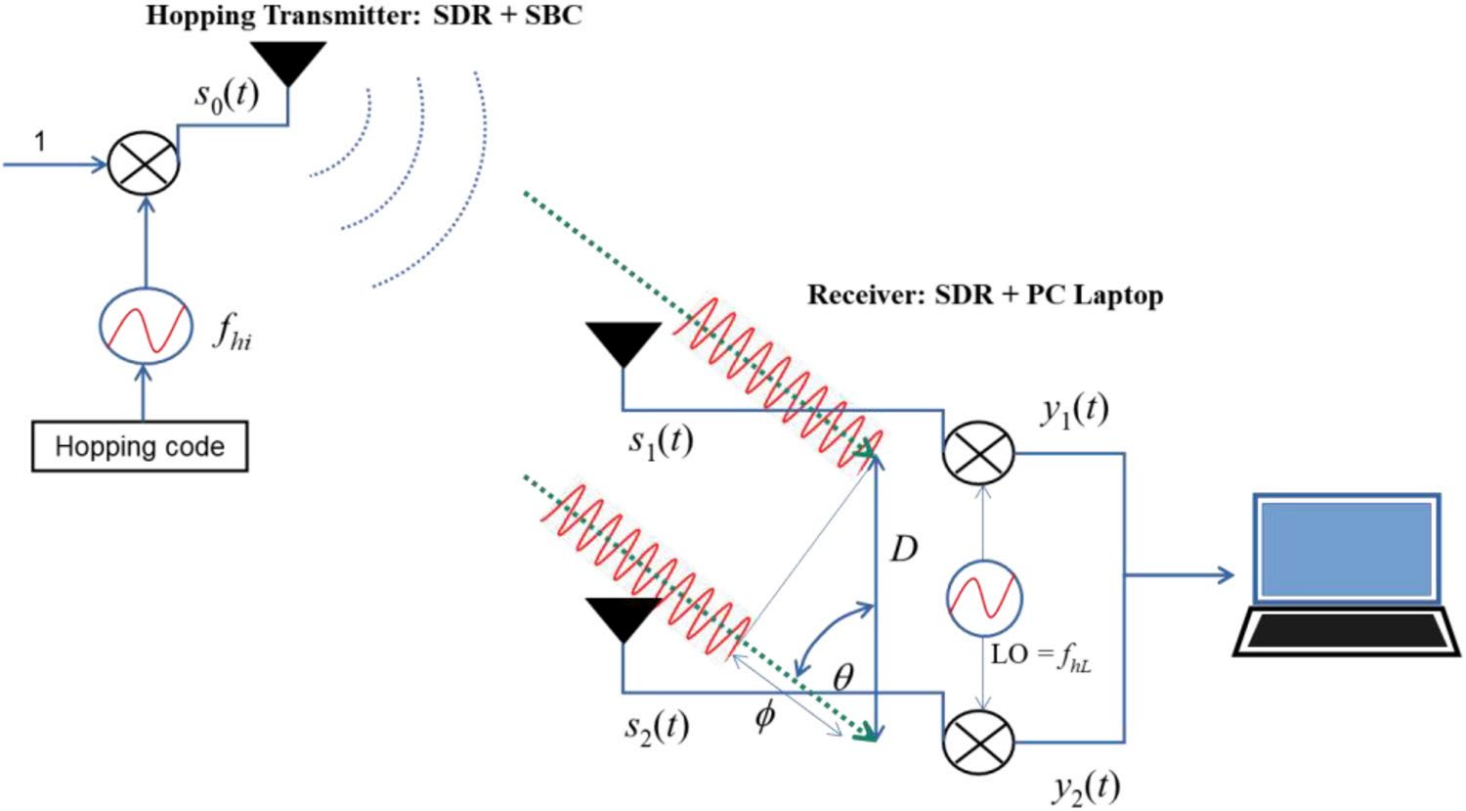
A system for tracking the AOA of stationary or moving cooperative objects.

This section addresses the fundamental elements of the proposed technique for estimating the AOA. Firstly, Interferometry refers to a method that amplifies the precision of azimuth angle determination by examining the interference patterns generated from multiple antennas. This method measures the phase difference between received signals, allowing for more precise azimuth angle determination. Interferometry offers high accuracy, providing a high angular resolution and accurate measurement of angles. Moving on, the cooperative system designed to track azimuth angles comprises of two essential components: a hopping transmitter positioned on the object and a receiver located at the monitoring station. The receiver is equipped with two antennas, as illustrated in Fig. 1. For this study, two SDRs were utilized as transmitters. These SDRs were controlled by a single-board computer (SBC). On the other hand, the SDRs operated as receivers and were controlled by a Personal Computer (PC).

The object was equipped with a transmitter that was preprogrammed to emit an uninterrupted transmission (known as continuous wave or CW) that alternates its carrier frequency. The hopping signal, which is transmitted for the purpose of measuring the AOA between two antennas, plays a crucial role. The AOA denotes the direction from which a signal arrives at a receiving antenna. To achieve this, a well-known signal is emitted and subsequently received by the antenna receiver. Within the Analog to Digital Converter (ADC) sampling capability in a specific frequency band, the transmitter carrier signal undergoes a distinctive frequency hopping modification. In situations involving multiple transmitters, distinct frequency hopping values are assigned to each detected object for identification purposes. By examining the frequency phase of the carrier signal, the AOA angle is ascertained. The signal emitted by the transmitter was described using the following representation:

1 $${s}_{0}\left(t\right)={a}_{0}{e}^{j\left(2\pi {f}_{hi}t\right)}$$

where ${a}_{0}$ was the amplitude and ${f}_{hi}$ was the hopping carrier. In order to capture the signal emitted by the transmitter, the receiver requires a sampling signal, which is generated by an ADC, with a frequency range that is at least twice the difference between the highest (${f}_{hL}$) and lowest (${f}_{hH}$) hopping frequencies. Consequently, the extent of the hopping frequencies is restricted to facilitate detection, and this can be achieved using the subsequent expression:

2 $${f}_{hL}<{f}_{hi}<{f}_{hH}$$

**Figure 2:**
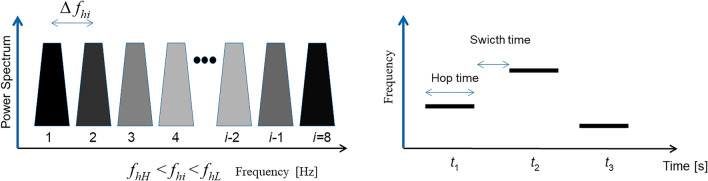
Range of frequency-hopping on the transmitter.

In this study, the frequency-hopping transmitter is denoted as ${f}_{hL}$ for the lower limit and ${f}_{hH}$ for the upper limit. Figure 2 presents the frequency-hopping signal model used in this research. A specific frequency sequence [*f*_*h*1_, *f*_*h*2_, *f*_h3_,…, *f*_*hi*_] determines the hopping frequency value for a detected cooperative object, where *f*_*hi*_ represents the *i*-th random hopping frequency. Here, *i* denotes the number of random frequencies employed as hopping frequencies for a specific object. Initially, *i* pseudo-random sequences are generated and utilized as the selection order. If a sequence has already reached its *i*-th value, the process restarts from the beginning. When multiple objects are detected, each object is assigned a unique frequency sequence to guarantee that the hopping frequency value is distinctive for every cooperative object.

The following is the expression used to represent the transmission of a signal emitted by a cooperative object, which travels a specific distance and is subsequently received by the initial receiver antenna:

3 $${s}_{{1,2}}\left(t\right)={a}_{{1,2}}{e}^{j\left(\left(2\pi {f}_{hi}+{f}_{d}\right)\left(t-R/C\right)+{\phi }_{{1,2}}\right)}+{n}_{{1,2}}\left(t\right)$$

where, ${a}_{1}$ and ${a}_{2}$ represents amplitude at the antenna 1 and 2. ${f}_{d}$ represents the frequency shift caused by the movement of an object relative to the transmitter. $R$ denotes the separation between the transmitting and receiving antennas. $C$ symbolizes the velocity of the radio wave, and ${\phi }_{{1,2}}$ signifies the alteration in phase. ${n}_{{1,2}}\left(t\right)$ symbolizes random noise at the antenna 1 and 2. When the cooperative object ceases its motion, the Doppler frequency ${f}_{d}$ becomes null. Additionally, the frequency of the Local Oscillator (LO) at the receiver is represented as follows:

4 $$LO={f}_{hL}$$

In order to guarantee the synchronization of data samples recorded at the PC receiver, receivers 1 and 2 in the SDR system shared the LO. The mathematical representation of the IF (intermediate frequency) signal at the receiver is as follows:

5 $${y}_{{1,2}}\left(t\right)={a}_{{1,2}}{e}^{j\left(2\pi \left({f}_{hi}+{f}_{d}-{f}_{hL}\right)\left(t-R/C\right)+{\phi }_{{1,2}}\right)}+{n}_{{1,2}}\left(t\right)$$

The difference in phase between the two signals, referred to as $\Delta \phi$, was computed by subtracting the phase of the signal obtained by the first antenna, known as ${\phi }_{1}$, from the phase of the signal obtained by the second antenna, referred to as ${\phi }_{2}$. In order to ascertain the hopping frequency of the received signal, the IF signal was converted into its respective frequency spectrum using the Discrete Fourier Transform (DFT) algorithm. This mathematical representation involves *N* samples of $n$ and can be expressed as follows:

6 $${Y}_{1,2}\left(k\right)={\sum }_{n=0}^{N-1}{y}_{{1,2}}\left(n\right){e}^{-j\frac{2\pi kn}{N}},\quad k={0,1},\dots,N-1$$

where, $k$ represents frequency index. In the course of our experiment, the FFT algorithm was employed for computing instead of the DFT. This application of the FFT algorithm significantly enhanced the speed of data processing. This was made possible by leveraging the built-in FFT capabilities provided by GNU-Octave. To identify the most prominent frequency present in the signal, the peak detector algorithm was employed. If this frequency corresponds to the frequency-hopping of the cooperative object, the AOA estimation was proceeded. Next, the signal underwent further processing using a bandpass filter that was made use of a Fourier filter. To ensure the elimination of any interfering signals, the center frequency was adjusted to the Fourier filter to match the detected hopping frequency. A Fourier filter is a filtering technique that enables manipulation of specific frequency components of a signal. Fourier bandpass filters provide several benefits, such as accurate frequency selectivity, ease of implementation, and adjustable parameter flexibility. These filters effectively isolate the desired frequency while efficiently eliminating unwanted frequency signals. Furthermore, Fourier bandpass filters display excellent computational efficiency, especially when utilizing the fast Fourier transform algorithm. This enables real-time processing and allows for the utilization of limited computing resources in various applications. In this process, the FFT of the detected hopping signal is taken, and certain frequencies are either attenuated or amplified selectively by convolving the original signal ${y}_{{1,2}}\left(t\right)$ with the Gaussian Fourier filter function $H\left(f\right)$ in the frequency domain. This manipulation can be expressed mathematically using the following equation.

7 $${X}_{{1,2}}\left(f\right)={Y}_{{1,2}}\left(f\right)H\left(f\right)$$

The mathematical expression for the Gaussian Fourier band-pass filter in the frequency domain was formulated as presented in a paper^26^:

8 $$H\left(f\right)=exp\left[-{\left(\frac{f-{f}_{hi}}{2\alpha }\right)}^{2}\right]$$

The given equation utilizes a Gaussian Fourier band-pass filter in the frequency domain using a mathematical expression called *f*_*hi*_, where the defined frequency-hopping is represented. The Gaussian parameter, $2{\alpha }$, is also incorporated. Simultaneously, the impulse response $h\left(t\right)$ is transformed into $H\left(f\right)$ using the FFT. To retrieve the signal output in the time domain, ${x}_{{1,2}}\left(t\right)$, the inverse FFT of ${X}_{{1,2}}\left(f\right)$ is applied.

After conducting the Fourier filtering process, two sinusoidal IF signals were acquired as the result. Numerous authors have put forth several algorithms to detect the phase disparity between these signals, one of which involves utilizing the Fourier algorithm. For our study, the FFT algorithm was opted, primarily due to its rapidity and simplicity. The Coarse Estimation FFT algorithm aids in the transformation of signals from the time domain to the frequency domain for the purpose of analyzing carrier signals. This process facilitates the identification of dominant frequencies and their associated angles at which signals are received by the antenna. Notably, it exhibits resilience against both additive noise and harmonic distortion. Moreover, it offers significant advantages in terms of processing speed. The phase difference between the two signals, obtained through the Fourier filtering process and computed by applying arc-tangent calculations on the ADC raw data, can be concisely expressed as follows:

9 $${\phi }_{{1,2}}=\angle \left\{{\sum }_{n=0}^{N-1}{x}_{{1,2}}\left(n\right){e}^{-j\frac{2\pi kn}{N}}\right\},\quad k={0,1},\dots,N-1$$

By referring to the elucidation presented in Fig. 1 and the accompanying equations, the AOA of the cooperative object can be approximated through the utilization of the phase difference, as delineated by the subsequent equation:

10 $${\theta }_{FFT}={sin}^{-1}\left(\frac{\lambda ({\phi }_{2}-{\phi }_{1})}{2\pi D}\right)$$

In this context, ${\theta }_{FFT}$ denotes the angle that is derived through the process of FFT calculation. The equation makes use of a value called $\lambda$, which represents the wavelength. Additionally, it takes into account the distance between the receiver antennas, denoted as *D*. It is important for this distance to be less than half of the value of $\lambda$ to avoid any ambiguity. Furthermore, specific frequency parameters associated with this technique have been conveniently presented in Table 1.

**Table 1:** Frequency parameters of different signals.

Signal	Frequency parameter
Transmitter frequency-hopping	${f}_{hL}<{f}_{hi}<{f}_{hH}$
Lower limit frequency	${f}_{hL}$
Upper limit frequency	${f}_{hH}$
Receiver LO	${f}_{hL}$
Doppler frequency	${f}_{d}$

### MUSIC algorithm

Schmidt introduced the MUSIC algorithm^27^ in 1979 with the aim of distinguishing between signals and noise by leveraging the orthogonal characteristics of signals in space. This was achieved through the eigen decomposition of the correlation matrix of the received signal. In the scope of the present investigation, the antenna array *M* consisted of 2 elements, the separation between the elements was described as *D*, the number of sources was denoted by ${s}_{1}$, and the incoming signal was assumed to originate from a single direction. The total number of signals can be determined or estimated by utilizing different methods, as described in reference^28^. The leading eigenvectors from the signal subspace (*Q*_*S*_) were distinguished from the remaining eigenvectors, which corresponded to the noise subspace (*Q*_*N*_). The pseudo-spectrum of the MUSIC algorithm demonstrated orthogonality between the noise and signal subspaces.

11 $${P}_{MUSIC}(\theta )=\frac{1}{{a}^{H}(\theta ){Q}_{N}{Q}_{N}^{H}a(\theta )}$$

The highest point of ${P}_{MUSIC}$ was regarded as the AOA of the signal. The AOA angle ranged from − 90° to + 90°. In order to minimize the search time, the ${\theta }_{e}$ constraint can be applied, which represents the most substantial error derived from the simulated RMSE estimation.

12 $${(\theta }_{FFT}-{\theta }_{e})<{\theta }_{sectoral}<({\theta }_{FFT}+{\theta }_{e})$$

To evaluate the precision of the estimated phase and AOA, RMSE was utilized. By comparing the estimated values with the actual values using Eq. (12), the results under varying SNR and angle fluctuations can be analyzed^29,30^.

13 $$RMSE\left[\phi \right]=\sqrt{\frac{\sum ({\phi }_{\text{obs}}\left[n\right]-{\phi }_{\text{sim}}\left[n\right]{)}^{2}}{N}}$$

In the meantime, the error associated with the AOA angle can be expressed in the following manner:

14 $$RMSE\left[\theta \right]=\sqrt{\frac{\sum ({\theta }_{\text{obs}}\left[n\right]-{\theta }_{\text{sim}}\left[n\right]{)}^{2}}{N}}.$$

**Figure 3:**
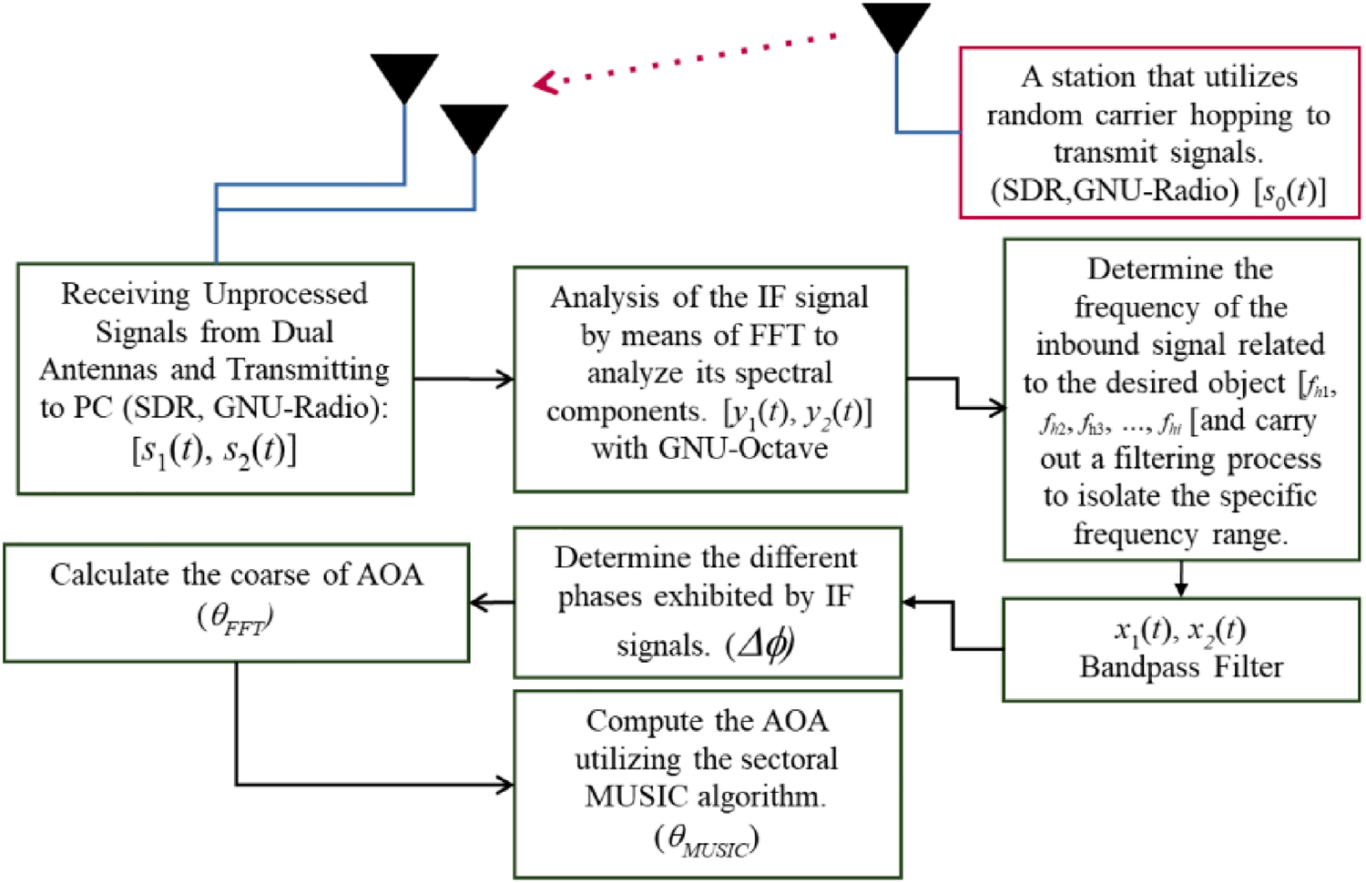
The diagram illustrating the AOA estimation algorithm for cooperative hopping transmitters.

The method proposed in this paper for estimating AOA is illustrated in a flowchart depicted in Fig. 3. The coarse-to-fine approach implemented in this graph was a progressive technique to enhance the accuracy of estimating the AOA. It follows a step-by-step process, commencing with an initial rough estimation using the FFT algorithm and gradually refining the measurement precision with the aid of the MUSIC algorithm. Although the coarse stage provides a speedy algorithm for generating a preliminary AOA, its accuracy is somewhat limited due to factors like noise, multipath interference, or restricted resolution. To address these limitations, the fine stage employs the MUSIC algorithm, which effectively mitigates the shortcomings of the coarse estimate and substantially improves the accuracy of AOA estimation. Recognized for its versatility and robustness, the MUSIC algorithm offers exceptional angular resolution and adaptability across various signal characteristics. It is well-suited for diverse sensor array configurations, encompassing radar, sonar systems, wireless communications, and antenna arrays. However, it is important to note that the computational complexity of the MUSIC algorithm can be demanding, particularly for extensive arrays and high-dimensional signal spaces. To reduce this computational time, a combination of the MUSIC algorithm and other algorithms such as FFT can be used in a coarse to fine method. This integration allows for accelerated processing speeds while maintaining the required accuracy. The GNU-Radio software was employed to facilitate the programming and control of the SDR at both the transmitter and receiver end. On the transmitter side, the software regulates the SDR hardware to transmit frequency-hopping signals randomly and continuously. On the receiver side, the software primarily performs data acquisition on a personal computer. In contrast, the GNU-Octave software is utilized for processing various algorithms proposed in this paper, such as filtering, phase calculation, and AOA angle estimation. The selection of these software packages is justified by their capability to execute essential functions like FFT, inverse FFT, convolution, peak detection, eigenvalues, eigenvectors, and other functions required for real-time data transfer from the SDR through a Transmission Control Protocol (TCP) connection. In order to validate the proposed method, besides simulations, experimentation using SDR was also conducted, which was thoroughly discussed in the subsequent section. The use of SDR technology plays a crucial role in evaluating the suggested angle of arrival interferometry approach. SDR facilitates the application of vital signal processing algorithms and techniques, enabling the simultaneous reception and processing of radio signals using multiple antennas. This adaptability allows for real-time signal processing and analysis, empowering researchers to comprehensively evaluate the effectiveness of the proposed methodology across different scenarios.

## Simulation and experiment results

In this section, the simulation findings regarding the influence of SNR on the precision of AOA calculations at a hopping frequency were assessed. Moreover, experimental results to substantiate and verify the simulation results were provided. Within this study, the phase-based AOA calculation at a hopping frequency was compared with the MUSIC algorithm. The simulation using the GNU-Octave software was carried out to accomplish this comparison. Furthermore, outdoor experiments employing SDR to validate the results and mitigate any potential effects caused by multipath interference were conducted. The process of outdoor testing was utilized to assess the effectiveness and precision of an AOA estimation system. This entailed the implementation of the system in real-life settings, examining data from numerous antennas, and taking into account various aspects including signal integrity, interference, and obstacles. The outcomes showcased the suitability of simulation and analysis findings across different environments, shedding light on potential enhancements as well as constraints. The simulation and experimental parameters or limitations for this study can be seen in Table 2.

**Table 2:** Simulated and experimental parameters.

Parameter	Values
Frequency-hopping carrier [${f}_{h1}$, ${f}_{h2}$, ${f}_{h3}$,…,${f}_{h(i=8)}]$	900 + [1.555, 2.555, 3.555, 4.555, 5.555, 6.555, 7.555, 8.555] MHz
One hopping time	12.5 ms
Switching time between hopping	138 μs
One hopping sequence time	100.97 ms
Receiver local oscillator (LO)	900 MHz
Lower limit frequency ${f}_{hL}$	900 MHz
Upper limit frequency ${f}_{hH}$	909 MHz
ADC receiver	30 MSPS
Base-line D	24 cm

### Simulation results

**Figure 4:**
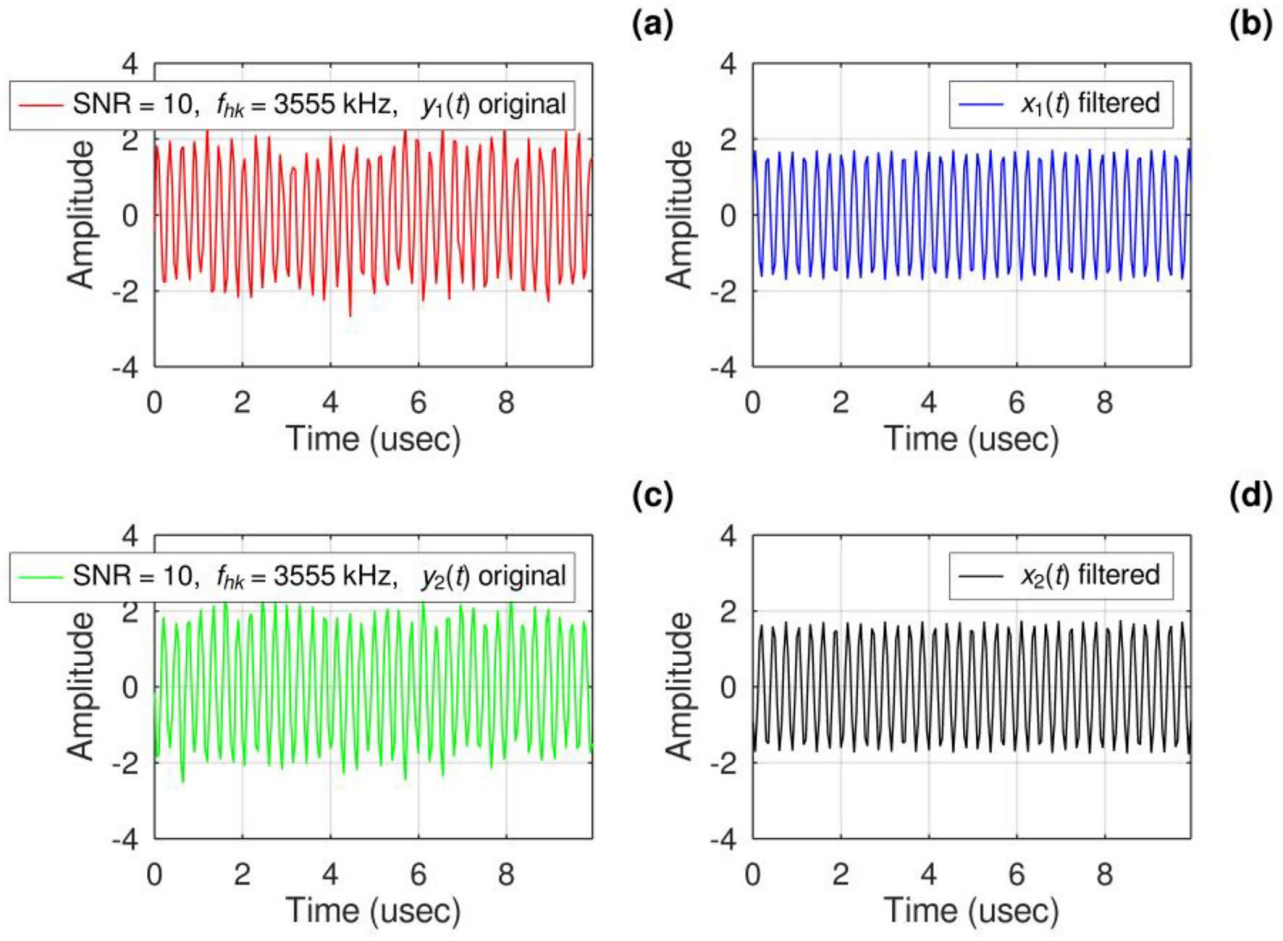
Simulation of the received signal at antenna 1 and antenna 2 when SNR = 10.

In Simulation 1, an examination was carried out to assess the disparity in AOA error between two algorithms: the phase difference-based algorithm and the classical MUSIC algorithm. The evaluation spanned a range of 0–90° and was performed under an SNR of 10 dB. Upon reviewing Fig. 4a and c, it becomes apparent that the signals received by antennas 1 and 2 were indistinguishable. After applying the Fourier Bandpass Filter, the improved signal condition is evident in Fig. 4b and d, allowing for further calculations.

**Figure 5:**
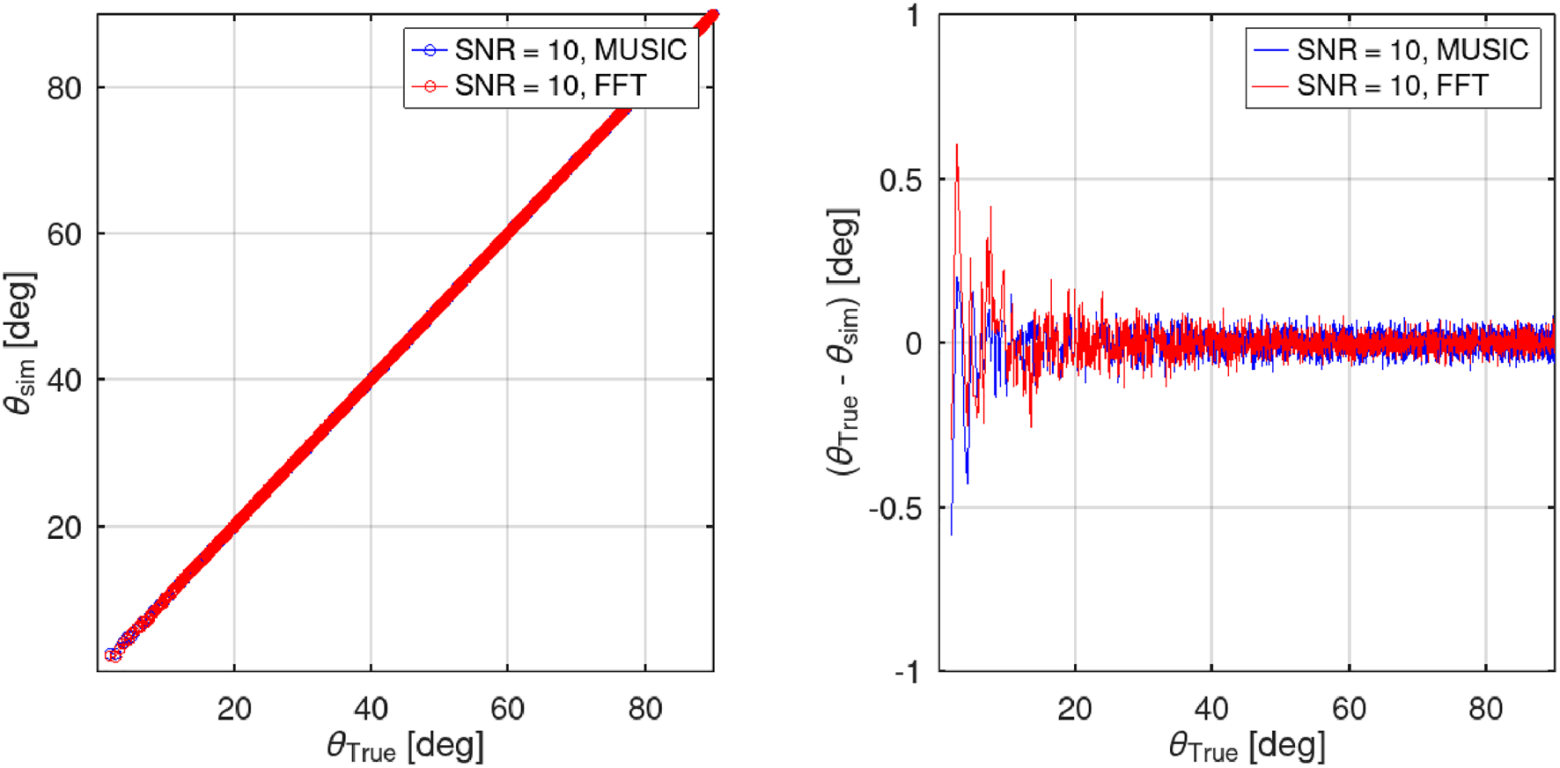
The simulation results exhibit the disparity in error measurements between the MUSIC algorithm and the proposed approach under a SNR of 10.

The results of the simulation, illustrated in Fig. 5, indicate that the calculation error of the AOA angle was most significant when the angle was near zero degrees. This situation corresponds to the scenario where the object was parallel to the receiver. Conversely, as the angle increases, the error diminishes gradually. Moreover, it can be noted that the degree of error exhibited by ${\theta }_{MUSIC}$ was typically half of that displayed by ${\theta }_{FFT}$.

**Figure 6:**
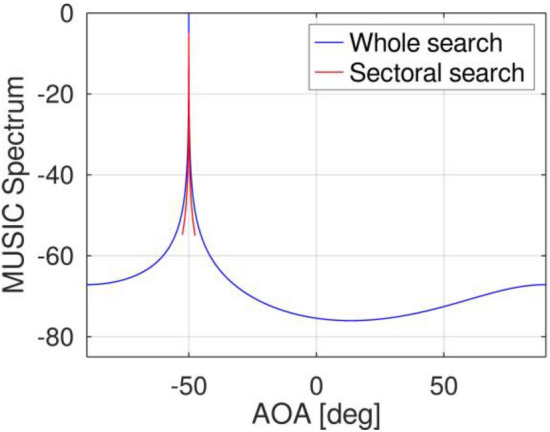
Conventional MUSIC and sectoral MUSIC algorithms utilize a narrower angle search range, limiting it to only *θ*_*e*_ = 2.5°.

In the present simulation, the error in estimating the AOA at different SNRs was analyzed. Employing the same experimental settings as Simulation 1, except for the SNR, the AOA estimation error across varying SNR values was evaluated. The tested SNR range spans from 0 to 30 dB, with an interval of 2 dB. To gauge the AOA error, the RMSE for both the phase and angle were calculated, utilizing 500 independent data points from the experimental trials. RMSE is a valuable metric for evaluating the performance of a particular method in terms of both accuracy and efficiency. It measures the average difference between predicted values and observed values in a given data set. Lower RMSE values indicate higher levels of precision and effectiveness. In this proposal, the scanning limits are determined using RSME as a benchmark. To ensure a fast process, the scanning limit is set at around 2.5°, which will be further explained in the next section of the simulation experiment. Initially, the AOA angle of the target signal was estimated using FFT. By employing Eq. (12) to calculate the angle from the FFT results, the range for the MUSIC angle search was determined. This approach enables a significant reduction in the MUSIC angle search range, as depicted in Fig. 6, resulting in a 20.69-fold increase in computational speed. Furthermore, by transforming the FFT AOA angle into the MUSIC AOA, twice the accuracy in the final angle estimation was achieved.

**Figure 7:**
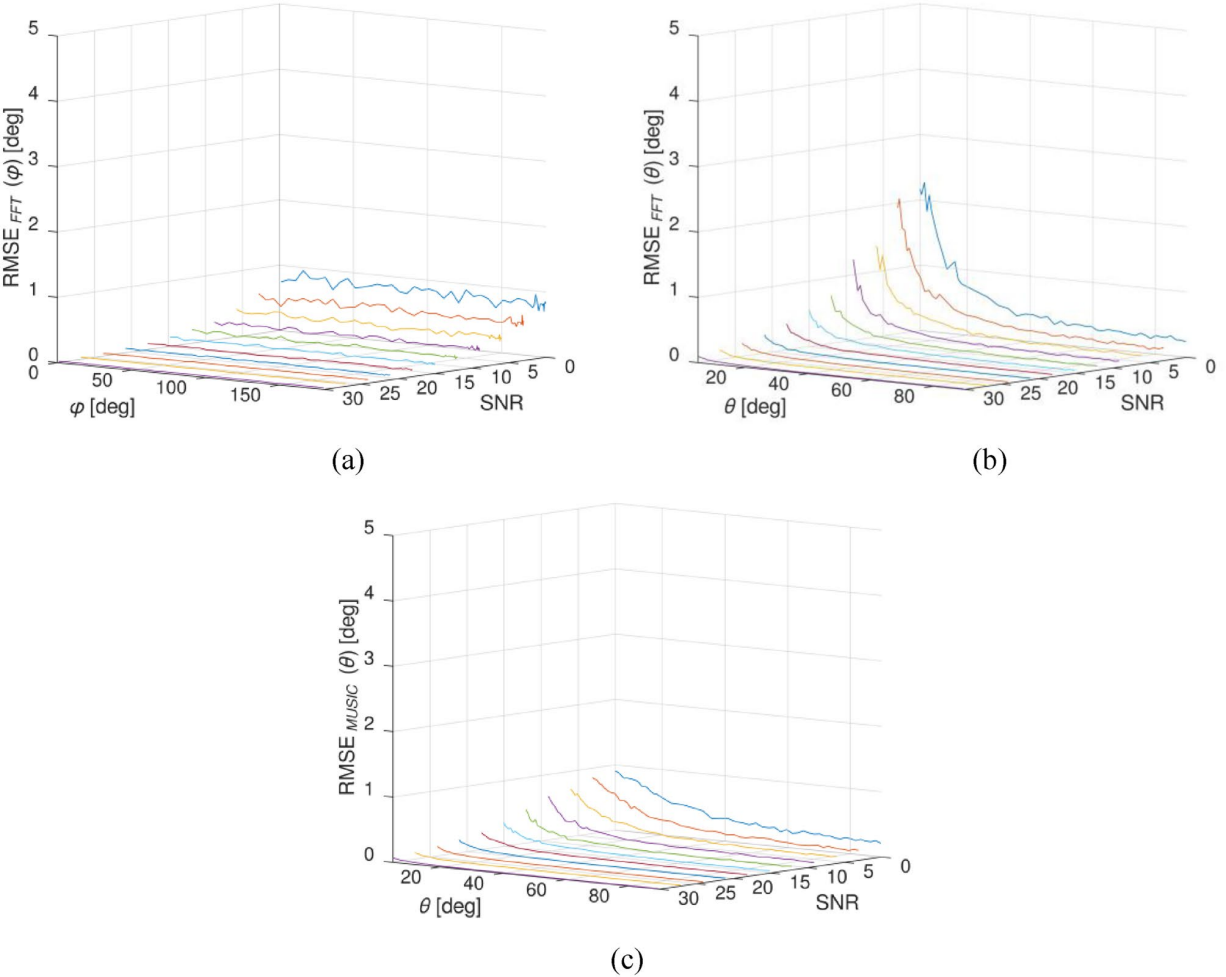
RMSE (**a**) for phase variation at various SNR, (**b**) for AOA estimation through FFT at different SNRs, and (**c**) for AOA estimation using the MUSIC algorithm at various SNRs.

The simulation results showcased in Fig. 7a reveal that the error in estimating the phase remains consistent across all phase angles ranging from 0 to *π*. It is noteworthy that when the SNR decreases, the error in phase detection increases. The analysis of Fig. 7a suggests that the RMSE value is primarily influenced by variations in SNR magnitude rather than the phase itself. However, upon examining Figs. 7b and c, it becomes evident that both changes in SNR and angle have an impact on the RMSE values. Figure 7b demonstrates the effect of varying SNR ratios on the error of AOA estimation. As the SNR drops, the estimation yields a relatively high error of 0.2° for an angle of 90°. Conversely, as the SNR increases beyond 20, the error diminishes to less than 0.2°. Moreover, as the AOA decreases, the error value rises, peaking at approximately 2.2° as the angle approaches zero. These specific error values serve as the threshold for the initial scanning range used in angle calculation through the implementation of the MUSIC algorithm. By utilizing this approach, one can attain an ideal equilibrium between the precision of precise estimation and the prevention of potential errors that may arise upon surpassing certain error thresholds. Consequently, it guarantees that the estimation procedure remains within acceptable limits and generates reliable outcomes.

The results of the third simulation, which examined the RMSE of AOA using the MUSIC algorithm, were presented in Fig. 7c. This simulation employed the error boundaries derived from the preceding simulation, which assessed the impact of the SNR on the accuracy of AOA calculation at different hopping frequencies. The results reveal that, under the lowest SNR condition, the maximum shift in RMSE utilizing the MUSIC algorithm was 1.0°. These findings unequivocally demonstrate the superiority of the MUSIC algorithm RMSE over that of the FFT. Consequently, based on these findings, it is suggested that the FFT be employed for initial angle scanning, whereas the actual angle calculation process should utilize the MUSIC algorithm. The technique used to detect signal direction in multi-source environments involves a combination of the Coarse-to-fine method, the FFT algorithm, and the MUSIC algorithm. By targeting distinct sectors, this algorithm selectively calculates the AOA while effectively eliminating undesirable signals and noise. This streamlined scanning approach significantly improves the precision of signal localization and separation, offering an enhanced level of accuracy. Moreover, compared to conventional methods, this approach enables a scanning process that is 18 times quicker, saving valuable time and resources. Based on the simulation results, it has been observed that employing ${\theta }_{sectoral}$ yields identical levels of accuracy as ${\theta }_{MUSIC}$ which improved rather than using accuracy of ${\theta }_{FFT}$, albeit achieving significantly faster processing speeds. Sectoral signal processing algorithms, in conjunction with high-speed ADC, robust digital signal processors, and high-speed interfaces, enable the attainment of optimal processing speed. The system efficiently analyzes information as a unified entity by employing the coarse-to-fine method, achieving a processing speed of 53.34 ms. Within this timeframe, the system diligently evaluates the incoming signal and accurately determines the AOA utilizing the FFT algorithm. Subsequently, the system seamlessly transitions to the MUSIC algorithm for further processing. The summarized results of these simulations have been conveniently presented in Table 3.

**Table 3:** Result of simulation comparison between conventional and sectoral MUSIC.

Parameter	${\theta }_{FFT}$	${\theta }_{MUSIC}$	${\theta }_{sectoral}$	
			${\theta }_{FFT}$	${\theta }_{MUSIC}$
Time	8.86 ms	918.18 ms	8.86 ms	44.38 ms
RMSE (maximum)	2.2°	1.0°	1.0°	

In this study, the fourth simulation focuses on investigating the impact of jamming on the detected signal of an object. The simulation incorporates a spot model, where the jamming is applied at a predetermined frequency. Given that the Fourier filter employed has a range of 1 kHz from the IF, this simulation aims to evaluate how the system reacts to jamming frequencies that are both within and outside the Fourier filter range. Notably, the angle estimation results remain unaffected by the amplitude and phase values at the jamming spot occurring outside the Fourier filter, as the utilized filter is primarily based on frequency. If interference occurs within the Fourier filter range, the amplitude and phase characteristics of the interference may impact the ${\theta }_{MUSIC}$ value. Nevertheless, since the suggested model utilizes a hopping frequency, there was an opportunity to explore different angles at varying hopping frequencies and minimize the effects of interference.

**Figure 8:**
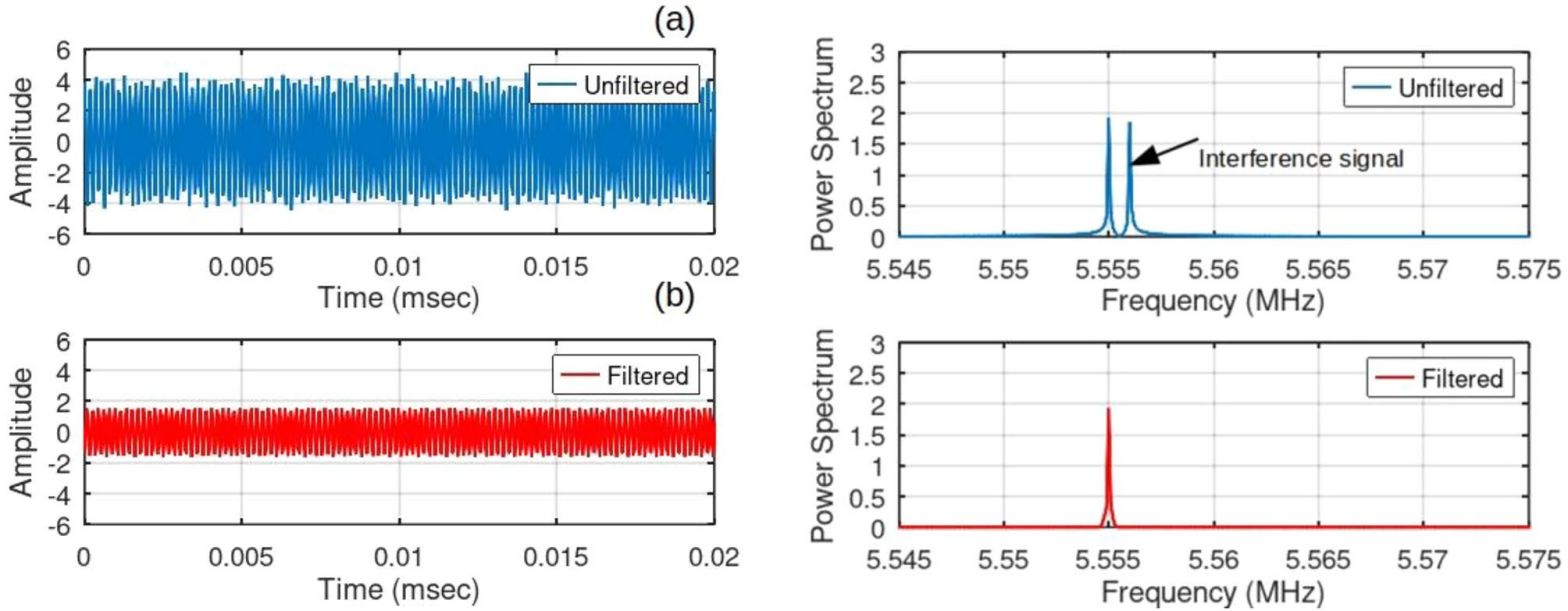
(**a**) Signal and spectrum with the jamming frequency, (**b**) Filtered signal and spectrum using a Gaussian Fourier filter.

In Fig. 8a, there was an image of a signal that shows the presence of a frequency interference signal. However, this interference signal can be efficiently removed by utilizing a Fourier Bandpass filter, as illustrated in Fig. 8b. The result of this simulation emphasizes the effectiveness of the suggested method in successfully handling jamming and frequency interference. Thus, it ensures the preservation of the desired signal integrity.

### Experiment results

**Figure 9:**
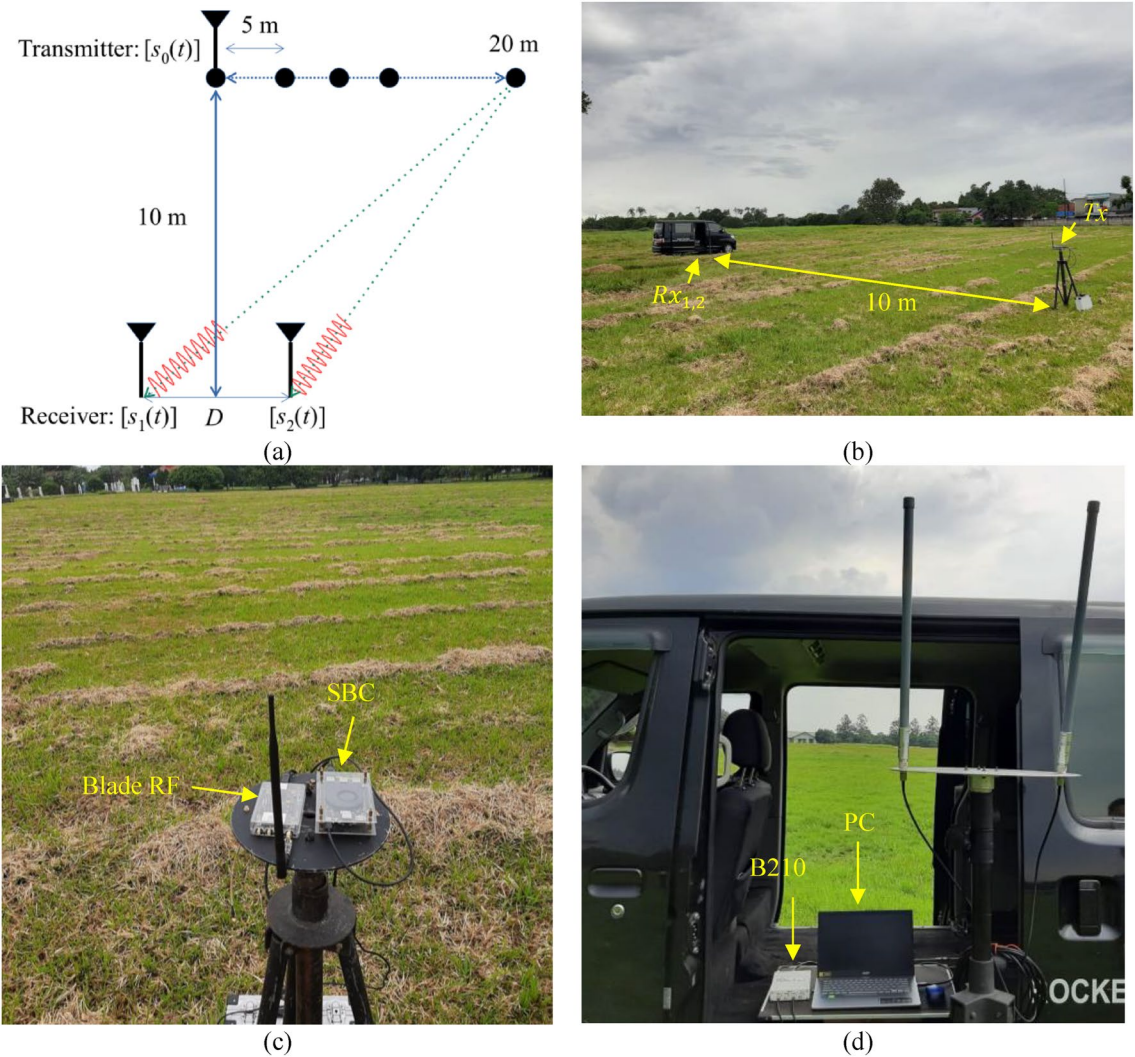
Experimental setup (**a**) the layout of transmitter and receiver positions outdoors. (**b**) Transmitter and receiver devices (**c**) transmitter devise with Nuand Blade-RF A90 and SBC Lattepanda Alpha. (**d**) receiver devise with USRP B210 and PC.

The trigonometry algorithm was employed to determine the distance between the transmitter and the receiver. The transmitter position was gradually shifted horizontally at an interval of 5 m until a distance of 20 m was achieved. The experiments were carried out in an outdoor setting, adhering to the transmitter and receiver positions illustrated in Fig. 9a. The implementation in the field followed the guidelines depicted in Fig. 9b.

The system employed a Nuand BladeRF as the transmitting device and utilized the USRP B210 from Ettus as the receiving device. In order to minimize interference from multipath effects, efforts were made, although reflections from the ground grass were still present. The transmitter in this particular experiment, as shown in Fig. 9c, made use of a Nuand BladeRF × 90 SDR transmitter, which was enhanced with an additional amplifier. It operated smoothly with the GNU-Radio software. The experimental setup for the transmitter can be observed in Fig. 1, which includes an SBC (Lattepanda Alpha) running on Linux Ubuntu, an SDR, and an omnidirectional antenna. The Nuand BladeRF xA9, employed as the transmitting device, was a sub-6 GHz device featuring a tunable phase-locked loop (PLL)-controlled LO. The transmitter frequency was randomly altered between the range of 900 MHz to 909 MHz, using the optional XMLRPC block of the GNU-Radio software. This provided the flexibility for a separate Python script to adjust the GNU-Radio LO frequency while the program was actively running.

Table 4 provides a comprehensive overview of the specifications pertaining to SDR and the antenna. For this experiment, the USRP B210 was utilized as the receiver owing to its capability of synchronizing devices and establishing a unified node employing two antenna arrays to function as receivers. Figure 9d illustrates the receiver setup in a detailed manner. The USRP B210 SDR was specifically selected due to its high-speed ADC, enabling the detection of variations in hopping frequencies. Before proceeding with any further experimentation, it was imperative to calibrate the received signal in order to synchronize the delays of the two received signals by utilizing an antenna splitter.

**Table 4:** Detailed Specifications of SDR USRP B210 and BladeRF.

Parameters	Values	
	USRP B210	Nuand BladeRF
Frequency range	70 MHz–6 GHz	47 MHz–6 GHz
ADC sample rate	61.44 MS/s, 12 bits	61.44 MS/s, 12 bits
DAC (Digital to Analog Converter) sample rate	61.44 MS/s, 12 bits	61.44 MS/s, 12 bits
Frequency accuracy	± 2.0 ppm	± 1.0 ppm
Power output	+ 10 dBm	+ 8 dBm
Antenna	Omnidirectional	Omnidirectional
Frequency range	900–930 MHz (RX)	700–2600 MHz (TX)
VSWR	≤ 1.5	≤ 1.5
Gain	4.5 dBi	5.0 dBi

**Figure 10:**
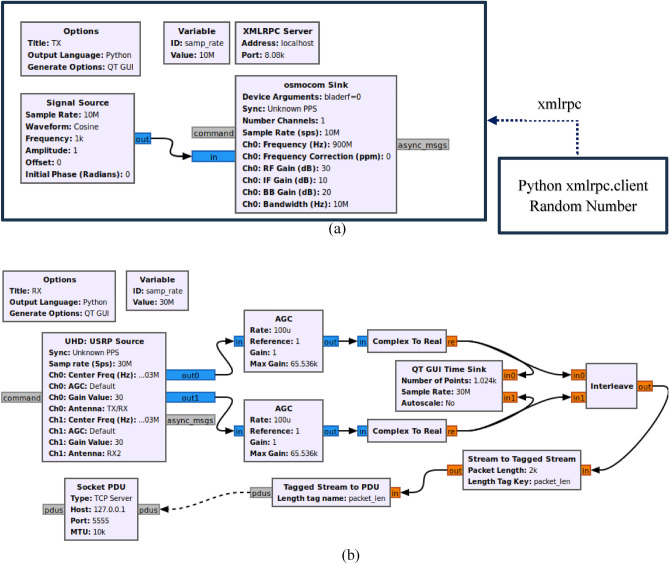
Two distinct GNU-Radio setups: (**a**) a transmitter system comprised of the Nuand BladeRF A90 device connected to the Lattepanda Alpha board, and (**b**) a receiver configuration consisting of the USRP B210 device linked to a PC.

The experiment incorporated a software system that comprised two main components, namely the transmitter and the receiver sections. The transmitter section employed a GNU-Radio program, depicted in Fig. 10a, to establish the central frequency at 900 MHz and subsequently adjust it based on the hopping value specified in Table 2. This specific block program was implemented via a Python script and executed by the Latte Panda Alpha. Contrarily, the receiver section employed a GNU-Radio block program, shown in Fig. 10b, to acquire data from the antenna utilizing SDR. At the same time, GNU Octave was used to process the acquired signal and convert it into an angle value. The connection between these two software applications involved employing the TCP/Internet Protocol (IP), which allowed for real-time retrieval and processing of data. It should be noted that the processing time required for this TCP/IP connection was taken into consideration as an additional factor when determining the overall acquisition time.

**Figure 11:**
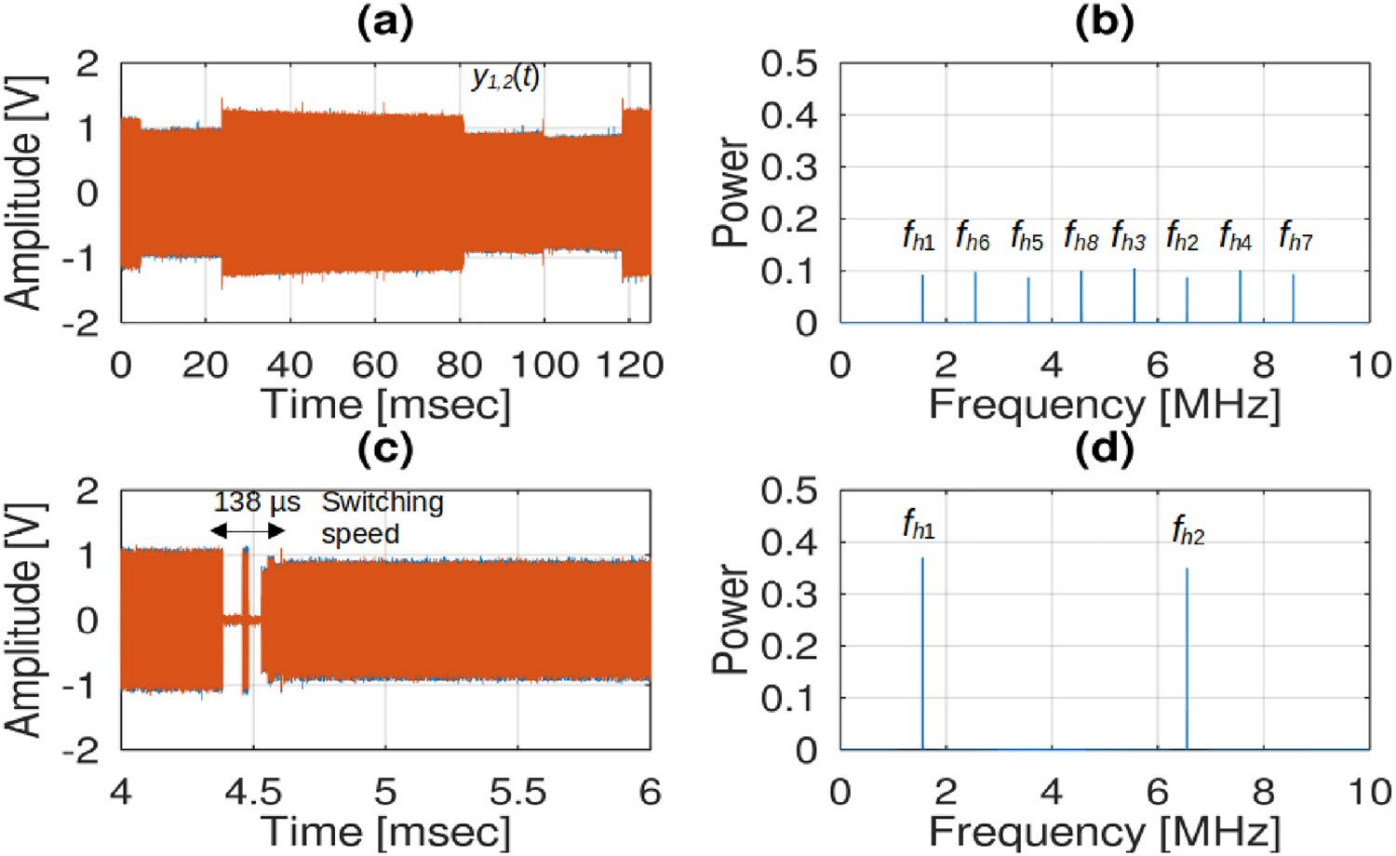
Signal ${y}_{1}\left(t\right)$ and ${y}_{2}\left(t\right)$ (**a**) 120 ms signal, (**b**) FFT of the signal, (**c**) 12.5 ms signal with switching speed, and (**d**) FFT of the 12.5 ms signal.

In order to accomplish the process of frequency hopping, the frequency of the LO was randomly modified among a total of eight frequencies, as shown in Fig. 11a and b, which are detailed in Table 2. Based on the results obtained from experimentation, as depicted in Fig. 11c, it is apparent that the time taken for the LO frequency to switch was measured to be 138 μs, while the corresponding time for completing one frequency hop was 12.5 ms. The XMLRPC block, which necessitates communication time with GNU-Radio, had an impact on the hopping speed and switching time. Consequently, there were approximately 79 hopping frequencies per second. Consequently, within one second, the hopping frequency could be repeated 10 times. It has been mentioned that the maximum hopping rate differs for various types of SDR, specifically the SDR B210 and Blade-RF, due to their integrated RF front ends that are not optimized for rapid hopping, as reported in^28^. By acquiring data for a duration of 12.5 ms, as depicted in Fig. 11c, it becomes feasible to record up to two distinct signals with varying frequencies. The FFT outcomes of the signal are presented in Fig. 11d, which reveals two prominent frequency peaks. Hence, the frequency corresponding to the largest number of sampled signals was selected for additional analysis in order to obtain the estimated AOA value.

**Figure 12:**
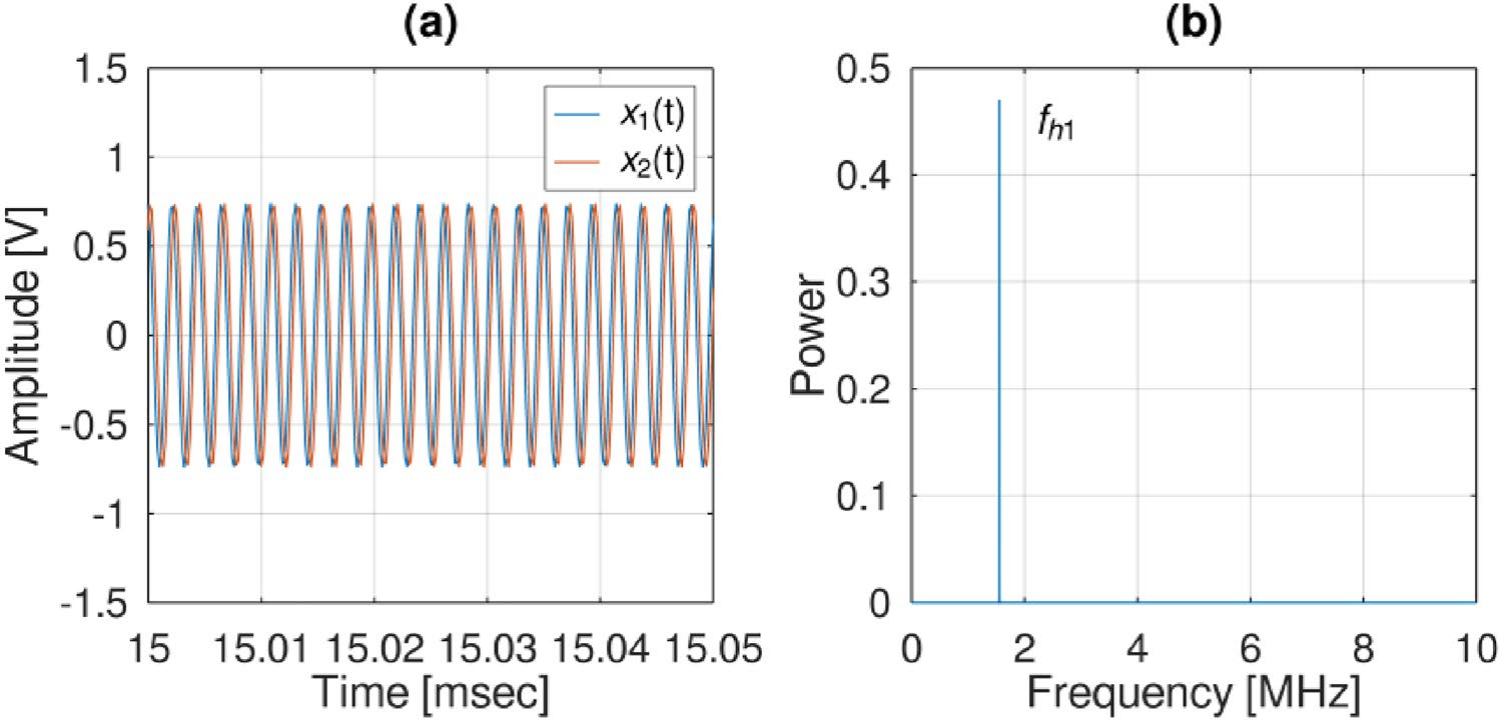
The IF signal obtained from two antennas following the application of a Gaussian bandpass filter, serving the purpose of estimating the final AOA, (**a**) signals, (**b**) power spectrum.

Figure 12 shows the representation of the IF signal obtained from two antennas after the application of a Gaussian bandpass filter. The suggested method employs a Gaussian Fourier filter to improve accuracy and reduce noise within a specific frequency range. This filter, founded on the Gaussian distribution and Fourier transform, operates as a bandpass filter by enabling frequencies within the designated range to pass through and diminishing those outside it. As a result, this technique guarantees an exact depiction of hopping signals and the seamless detection of such signals. This depiction serves the purpose of aiding the determination of the final estimation of AOA (a). Additionally, the strength spectrum of this signal is depicted visually (b). Throughout 12.5 ms, the hopping signal received was consistently recorded. The volume of data processed per second was taken into consideration for accurately estimating the AOA. It was ensured that each snapshot contained a single frequency hopping event. Subsequently, the signal was subjected to a Gaussian Fourier filter to pinpoint the initial angle calculation using the ${\theta }_{FFT}$ Eq. (14). Upon determining the ${\theta }_{FFT}$ angle, the ${\theta }_{sectoral}$ value was computed with equation (12), leading to a reduced scanning range for the MUSIC algorithm and thereby resulting in faster calculation time.

The results obtained from experiments carried out in an environment with an average SNR of 10 dB are elucidated in Table 5. This table elucidates that the error in the estimation of the AOA increases with the decreasing angle, reaching a maximum error shift of 1.34° when the angle approaches 0°. Conversely, as the angle approaches 90°, the error value decreases by approximately 0.2°. Analyzing the data presented in the table leads to the conclusion that the optimal angles for effective detection fall within the range of 45° to 90°. Consequently, it is recommended to configure the angle settings for detection accordingly.

**Table 5:** AOA error comparation between actual degree and experiment results.

Actual degree (°)	SNR (dB)	Experiment result (°)			
		${\theta }_{FFT}$	RMSE ${\theta }_{FFT}$	${\theta }_{MUSIC}$	RMSE ${\theta }_{MUSIC}$
26.56	9.3	25.698	1.339	25.948	1.306
33.69	9.5	33.691	1.003	33.441	0.851
45.00	9.8	44.765	0.569	45.015	0.519
63.43	10.2	63.387	0.473	63.137	0.304
90.00	10.4	89.855	0.183	89.898	0.158

The results of the conducted experiments in this study have proven that the suggested phase-based calculation method for AOA at hopping frequency is both precise and efficient. By employing the capability of FHSS and effectively utilizing a combination of hopping frequencies alongside with Gaussian Fourier filter, the optimal results can be obtained. This approach not only minimized interference but also enhanced processing speed while maintaining a high level of accuracy. The RMSE values from the experiments, as depicted in Table 5, indicate that the method achieves a maximum deviation of 1.30° at an angle of 26.56°. As the angle approaches 90°, the error diminishes to approximately 0.16°. Additionally, the proposed method, employing the Fourier filter, effectively withstands interference and surpasses other methods^31^ that employ the MUSIC algorithm without initial scanning in terms of speed. It enhances the precision and sharpness of spatial details, thereby enabling a wide range of applications such as accurately determining radio channel attributes, precise indoor positioning, monitoring the movement of objects, and identifying sources of interference. These capabilities are dependent on various factors, including the method of implementation, hardware specifications, and the techniques employed for signal processing.

In general, this proposed approach presents a promising method for calculating AOA in real-time systems like autonomous vehicles, RADAR systems, and Internet of Things (IoT) applications. There are potential areas of exploration pertaining to AOA within specific sectors and frequency hopping. These areas include multi-user detection and integration with IoT technology. In the case of tracking Alzheimer's patients outside^32^, the AOA frequency hopping method proves to be valuable. By employing this technique, it becomes possible to determine the direction of the signal emitted from either a wearable device or a GPS tracker. This information greatly assists in estimating the location of Alzheimer's patients, enabling caregivers and healthcare professionals to effectively monitor their movements and ensure their safety. It is essential to recognize that the accuracy and reliability of any tracking method, including AOA, can be impacted by a variety of factors such as environmental conditions, signal strength, hardware limitations, and system design. Therefore, a thorough assessment and testing process are imperative to evaluate the effectiveness of implementing these methods in real-life situations.

## Conclusion

This paper introduces a novel approach to estimating the AOA in frequency-hopping scenarios. The AOA estimation method proposed in this study effectively overcomes the negative impact of jamming on angle measurement by utilizing frequency-hopping. The Gaussian Fourier filter is employed to reduce noise within the chosen frequency range and to improve accuracy further. Moreover, the introduction of the sectoral AOA estimation concept enables a narrower scanning range for the MUSIC algorithm, resulting in a two-fold increase in accuracy. The system has the ability to detect in real time, achieving a speed of 53.34 ms for processing. The system specifically refers to the overall duration of the MUSIC and FFT sectoral procedures. Additionally, it successfully detects 18 angles per second. The utilization of the proposed sectoral AOA estimation method combined with frequency-hopping preserves the advantages of frequency-hopping while accurately determining the angle of a cooperative object.

## Data Availability

The authors confirm that the supporting data and material of this study are available from the corresponding author, upon reasonable request.
